# Projections of heat stress and associated work performance over India in response to global warming

**DOI:** 10.1038/s41598-020-73245-3

**Published:** 2020-10-07

**Authors:** K. Koteswara Rao, T. V. Lakshmi Kumar, Ashwini Kulkarni, Chang-Hoi Ho, B. Mahendranath, Srinivas Desamsetti, Savita Patwardhan, Appala Ramu Dandi, Humberto Barbosa, Sudhir Sabade

**Affiliations:** 1 Indian Institute of Tropical Meteorology, Ministry of Earth Sciences, Pune, India; 2 Atmospheric Science Research Laboratory, SRM Institute of Science and Technology, Kattankulathur, Tamilnadu India; 3 School of Earth and Environmental Sciences, Seoul National University, Seoul, South Korea; 4 Department of Meteorology & Oceanography, Andhra University, Visakhapatnam, India; 5 National Centre for Medium Range Weather Forecasting, Ministry of Earth Sciences, Noida, India; 6 Laboratorio de Analise e Processamento de Imagens de Satelites, Universiadade Federal de Alogoas, UFAL, Maceió, Brazil

**Keywords:** Climate sciences, Risk factors

## Abstract

Summertime heat stress future projections from multi-model mean of 18 CMIP5 models show unprecedented increasing levels in the RCP 4.5 and RCP 8.5 emission scenarios over India. The estimated heat stress is found to have more impact on the coastal areas of India having exposure to more frequent days of *extreme caution* to *danger* category along with the increased probability of occurrence. The explicit amount of change in temperature, increase in the duration and intensity of warm days along with the modulation in large scale circulation in future are seemingly connected to the increasing levels of heat stress over India. A decline of 30 to 40% in the work performance is projected over India by the end of the century due to the elevated heat stress levels which pose great challenges to the country policy makers to design the safety mechanisms and to protect people working under continuous extreme hot weather conditions.

## Introduction

Climate change, which is persuaded by the global warming effect, is a global concern due to its adverse impacts on various systems. The Fifth Assessment Report (AR5) of the Intergovernmental Panel on Climate Change (IPCC) findings confirm that the increasing anthropogenic greenhouse gas (GHG) concentrations which are responsible for the unusual Earth’s warming in recent decades^1^, cause the frequent high intensity heat extremes with prolonged duration affecting the living and working environments^2–5^. This increase in heat extremes will cause the damage to health, comfort and economic activities of millions of people across the globe^3,6^. It is reported that the increase in air temperature enhances the moisture holding capacity of the atmosphere that causes increase in heat stress due to the surface heating^6–8^. Many studies reported the heat stress by using only the temperature data without taking into account of humidity^9–11^. However, humidity plays an important role in discomfort due to warming and must be integrated to estimate the heat stress index^12–15^, particularly in tropical countries^16^. The study of heat stress is very important in the context of health impacts (eg: heat stroke) of the humans during their outdoor activities^17–22^. Extreme heat events lead to drastic decline in work productivity along with the heat related mortality^23–26^. Several methods/indices are used to estimate the heat stress using different environmental/meteorological factors. Some metrics use combinations of temperature and moisture, such as the the National Weather Service Heat Index and Humidex^27^, while others additionally include solar radiation (e.g. Wet bulb globe temperature (WBGT)^28^ and Environmental Stress Index^29^).

Unusual hot weather affects the work productivity of human societies by reducing their performance and increase in heat related illness^30–32^. Particularly, higher values of heat stress can cause deterioration in the work productivity of outdoor laborers where physically demanding tasks are required. Excessive heat stress increases the core human body temperature (above 37 degrees) thus, leading to heat stroke (severe hyperpyrexia), which causes the decline in the physical work capacity^33,34^. Increasing heat exposure is linked to occupational health risks and adversely impacts the work productivity^20,35^. Study of 370 onsite data sets in Hong Kong reported that the work duration of the construction labour has been decreased by 0.33% with one degree rise in WBGT^36^. Analysis of 8076 workers of 11 different studies revealed the 30% loses in work productivity due to rise in WBGT in the people who are working in single shift^37^. The loss in labour productivity due to heat stress over Australia has been reported^38^ and a decrease in productivity globally upto 20%. In tropical lands, the agricultural activities of working labor which involves higher levels of physical exertion had been badly affected by the heat stress^39^. It is reported that the estimated labour productivity in relation to heat stress index for 21 regions of the world infers a reduction in labour productivity due to warming of climate and resultant heat stress^40^. Also for the workers with low income in the tropical regions, heat stress is going to be the greatest health hazard where the facilities such as health surveillance are not available^41^. Venugopal^42^ studied the health and work productivity issues of the workers from 18 places in India and found that they are vulnerable to heat stress. The impact of heat stress on working people in hot environments is measured using WBGT by ISO 7243 standard^43^. Also, a few reports infer that the work ability estimated from the metrics of WBGT over New Delhi, India show better results compared to the other metrics such as Predictive Heat Stress, Universal Thermal Climate Index^44^. However, the studies of Brode et al.^44^ applied limited criteria in simulating the work ability during the day time working under light, moderate and heavy conditions and found the work ability change with *“work load, time of the day and climate type”*. Also, the results of the Brode et al.^44^ were suggested to be representatives of prevailing in agriculture,health and industrial sectors.

The present unusual warming is expected to increase faster and becomes vulnerable to physiological acclimatization among the workers carrying out activities in outdoor and indoor work places without thermal neutralities. Report from International Labour Organization (ILO) says the decline in global work productivity will be same as the loss of 80 million full time jobs by 2030 due to the increasing levels of heat stress (https://unfccc.int/news/international-labour-organization-warns-of-heat-related-job-losses). As India is more vulnerable to the heat extremes such as heat waves, heat stress due to its projected increasing temperatures^39,45–47^, knowing the impact of heat stress on the work performance helps the policy makers to take actions in order to protect the workers from the dangerous exposure of heat stress and to maintain the sustained work performance. Hence, it is important to assess the future changes in the heat stress index over India where in the outdoor activities are significant. The current study is built upon the assessment of heat stress along with its associated decline in work performance over India in the present and future climate under different emission scenarios. The results of the present work may be useful to the policy makers to design a better policy tools for taking necessary steps for the welfare of the working people due to the increased heat stress in future under changing climate conditions.

## Materials and methods

Here we used Steadman’s Heat Index (HI)^48^ approach which combines ambient air temperature and relative humidity to estimate the heat stress. Steadman's field observations and measurements were translated into a table that showed heat stress values and the corresponding risk levels (Table 1). On the basis of the table generated by Steadman, Rothfusz (1990)^49^ developed the following equation for heat index.

$$ \begin{gathered} {\text{HI }} = {-}{42}.{379 } + { 2}.0{49}0{1523}*{\text{T }} + { 1}0.{14333127}*{\text{RH}}{-}0.{22475541}*{\text{T}}*{\text{RH }} \hfill \\ {-} \, \left( {{6}.{83783} \times {1}0^{{{-}{3}}} *{\text{T}}^{{2}} } \right){-}\left( {{5}.{481717} \times {1}0^{{{-}{2}}} *{\text{RH}}^{{2}} } \right) \, + \, \left( {{1}.{22874} \times {1}0^{{{-}{3}}} *{\text{T}}^{{2}} *{\text{RH}}} \right) \, \hfill \\ + \, \left( {{8}.{5282} \times {1}0^{{{-}{4}}} {\text{T}}*{\text{RH}}^{{2}} } \right) \, {-} \, \left( {{1}.{99} \times {1}0^{{{-}{6}}} {\text{T}}^{{2}} *{\text{RH}}^{{2}} } \right) \hfill \\ \end{gathered} $$

where T is ambient dry bulb temperature (°F) and RH is relative humidity in percentage. This formula was obtained through a set of measurements, mathematically analyzed by multiple regressions. The values of HI are further converted in to Celsius scale and used in the present study. The categories of heat index are classified as (1) caution (27–32 °C) (2) extreme caution (32–41 °C) (3) danger (41–54 °C) and (4) extreme danger (> 54 °C) respectively^50^. More information on the evaluation of HI can be found in Supplementary Material.

**Table 1:** Categories of Heat Indices (HI), the Corresponding Risks Levels, and Heat Stress Classification, Along with the Health Problems.

HI (°C)	Risk levels	Classification	Health problems
27–32	Low	Caution	Fatigue possible with prolonged exposure and/or physical activity
32–41	Moderate	Extreme Caution	Sunstroke, muscle cramps, and/or heat exhaustion possible with prolonged exposure and/or physical activity
41–54	High	Danger	Sunstroke, muscle cramps, and/or heat exhaustion likely. Heat stroke possible with prolonged exposure and/or physical activity
> = 54	Very high to extreme	Extreme danger	Heat stroke or sunstroke is highly likely

A relationship is developed based on the experimental data sets of working performance^51^, thermal comfort^52^ and the decrement of work performance (P in %) has been found with the rise in temperature^53^. It is reported that the temperature and heat index values do not vary much in many areas^54^ and also the sensitivity of heat—health effect estimates are not showed larger differences to measure the exposure with heat index versus temperature^55,56^. Though there is a variation, particularly in summer time, a substantial correlation between these two infer the same quantitative results^54^. Also, when using the mean temperature, the indices such as heat stress index, humidex and relative strain index, the magnitudes of all do not vary much. Average temperature performed similarly to the composite indices, but minimum and maximum temperatures performed relatively poorer. Thus, average temperature may be suitable for the development of weather-health warming systems^55^. Hence in the present study, we have used the heat stress index instead of temperature since the heat stress is a better indicator than temperature in heat-health research.

Thus, the equation for estimating the decline in work performance can be written as:

$$ {\text{P }}\left( \% \right) \, = { 2 } \times \, \left( {{\text{Heat stress}}, \, ^\circ {\text{C}}} \right) \, {-}{ 5}0. $$

The mean of three reanalysis datasets viz, the ERA-Interim reanalysis from ECMWF^57^, NCEP-DOE-Reanalysis2^58^ and NOAA-CIRES 20th Century reanalysis^59^ for daily mean surface air temperature and relative humidity have been used for the estimation of HI. Daily simulations of 18 general circulation models (GCMs) data from suite of the Coupled Model Intercomparison Project 5 (CMIP5)^60^ (Table 2) and their Multi Model Mean (MMM) have been used to estimate the summertime (March to June) heat stress in the baseline period of 1986–2005 for the purpose of models evaluation. Also, we estimated the same for the three future time slices 2016–2035, 2046–2065 and 2080–2099 which are treated as near, mid and long term (IPCC, 2013)^1^ under the two emission scenarios of RCP 4.5 and RCP 8.5. All the models used in the present study have been interpolated to an uniform grid resolution of 1° × 1° using the bilinear interpolation method^61–63^ for making them consistent to compute the multi model mean and which will be helpful in studying the explicit diagnosis of heat stress on space and time scale.

**Table 2:** Details of the CMIP5 models used for the analysis.

S.No	Modeling center (or group)	Model name	Grid size
1	Commonwealth Scientific and Industrial Research Organization (CSIRO)	ACCESS1.0	192 × 145
2	Beijing Climate Center	BCC-CSM1-1	128 × 64
3	Beijing Climate Center	BCC-CSM1-1-M	320 × 160
4	College of Global Change and Earth System Science, Beijing, Normal University	BNU-ESM	128 × 64
5	Canadian Centre for Climate Modeling and Analysis	CanESM2	128 × 64
6	Centre National de Recherches Météorologiques	CNRM-CM5	128 × 256
7	Commonwealth Scientific and Industrial Research Organization	CSIRO-Mk3.6.0	192 × 96
8	NOAA Geophysical Fluid Dynamics Laboratory	GFDL-ESM2M	144 × 90
9	Met Office Hadley Centre	HadGEM2-CC	192 × 144
10	Met Office Hadley Centre	HadGEM2-ES	192 × 144
11	Institute for Numerical Mathematics	INM-CM4	180 × 120
12	Institut Pierre-Simon Laplace	IPSL-CM5A-LR	96 × 96
13	Institut Pierre-Simon Laplace	IPSL-CM5A-MR	144 × 143
14	Japan Agency for Marine-Earth Science and Technology, Atmosphere and Ocean Research Institute (The University of Tokyo)	MIROC-ESM	128 × 64
15	Japan Agency for Marine-Earth Science and Technology, Atmosphere and Ocean Research Institute (The University of Tokyo)	MIROC-ESM-CHEM	128 × 64
16	International Centre for Earth Simulation	MIROC5	256 × 128
17	Meteorological Research Institute	MRI-CGCM3	320 × 160
18	Norwegian Climate Centre	NorESM1-M	144 × 96

Matthews et al.^14^ and Lee and Brenner^64^ studied the global heat index, estimated from the temperature and relative humidity while Opitz-Stapleton et al.^65^ and Sylla et al.^19^ studied the same regionally. Lee and Berner^64^ reported that these heat stress indices are used by International Organization for Standardization (ISO) and National Institute for Occupational Safety and Health (NIOSH) to measure the heat loads in order to prevent the heat illness. These formulae need to be weighted by population density in order to study the impact on human health and productivity when they are subjected to study for different countries over the globe. During summer, the spatial variations of temperature are very less over India except in the regions of Himalayas and Northeast region of India, the heat stress levels and the decline in work performance have been taken into account over entire India except these two regions. Hence, the results obtained over Himalayan and Northeastern parts need not be considered in the present study.

## Results and discussions

**Figure 1:**
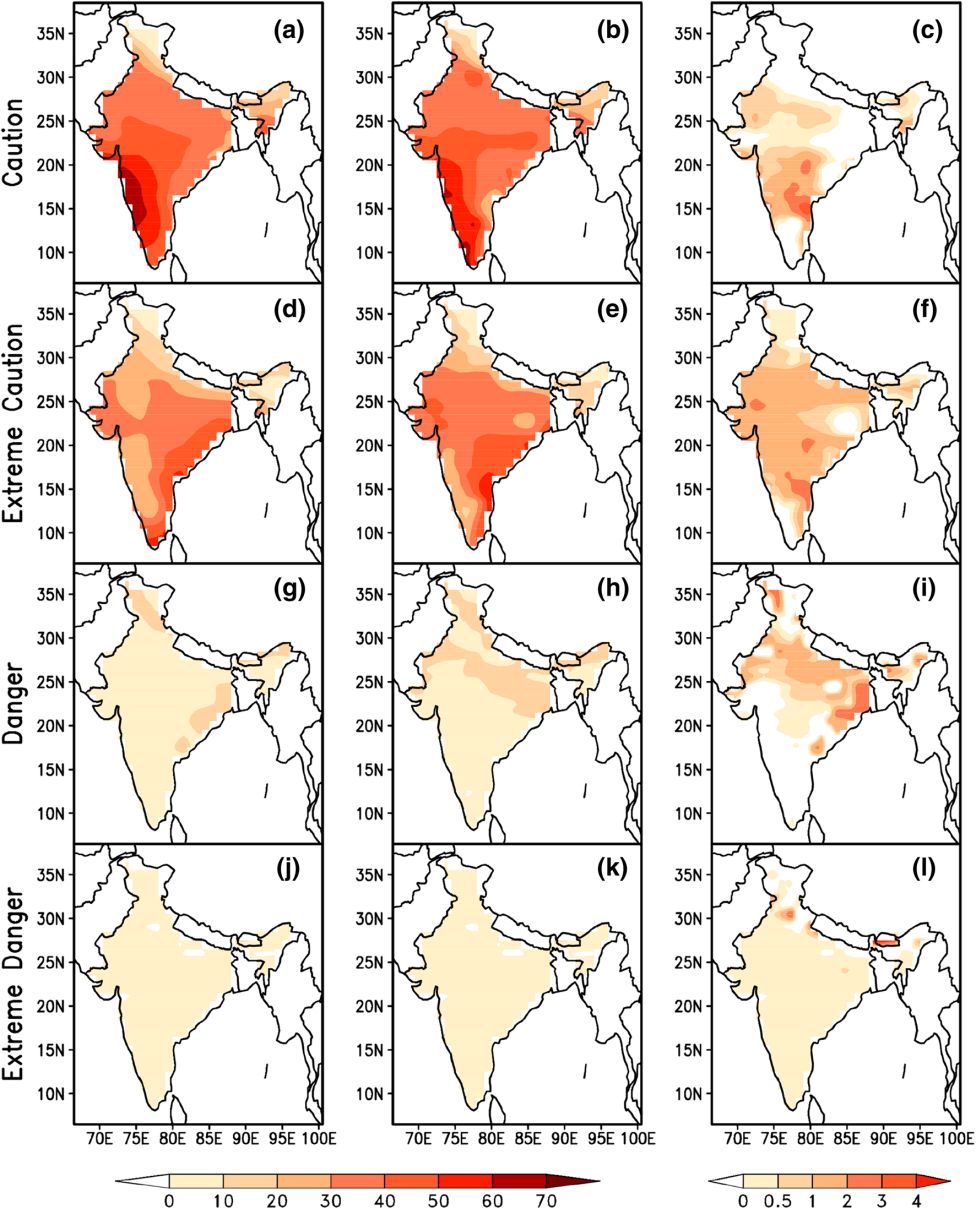
MMM of CMIP5 simulations (left column) of mean heat stress days (%) over India during summertime (MAMJ) compared with reanalysis (middle column) and the bias (right column) for four categories of heat stress. This figure has been generated using GrADs 2.1.1.6.0 https://cola.gmu.edu/grads.

**Figure 2:**
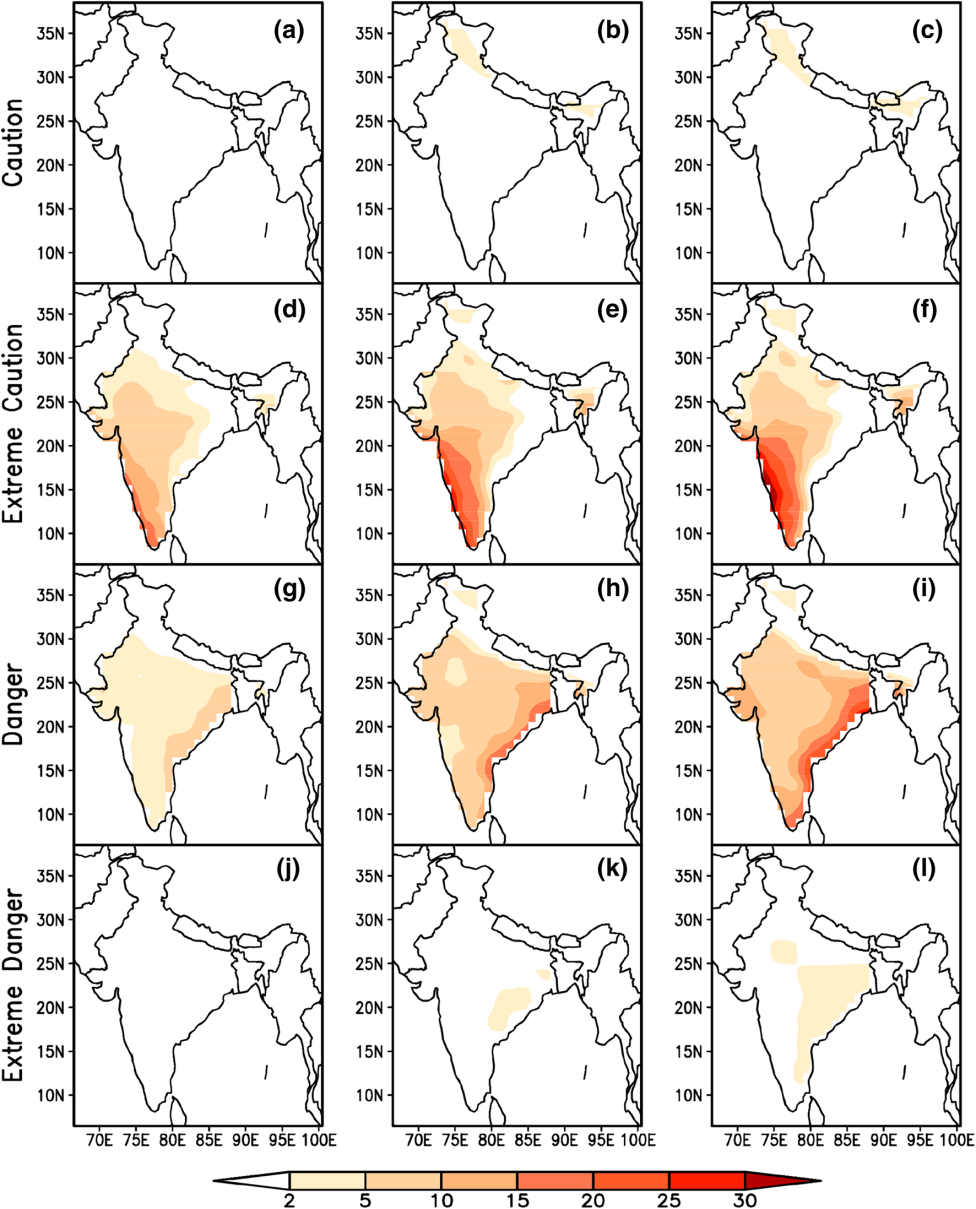
Projected changes in four categories of summertime heat stress days (%) under RCP 4.5, for three-time epochs 2016–2035 (left column), 2046–2065 (middle column) and 2080–2099 (right column) with respect to 1986–2005. This figure has been generated using GrADs 2.1.1.6.0 https://cola.gmu.edu/grads.

**Figure 3:**
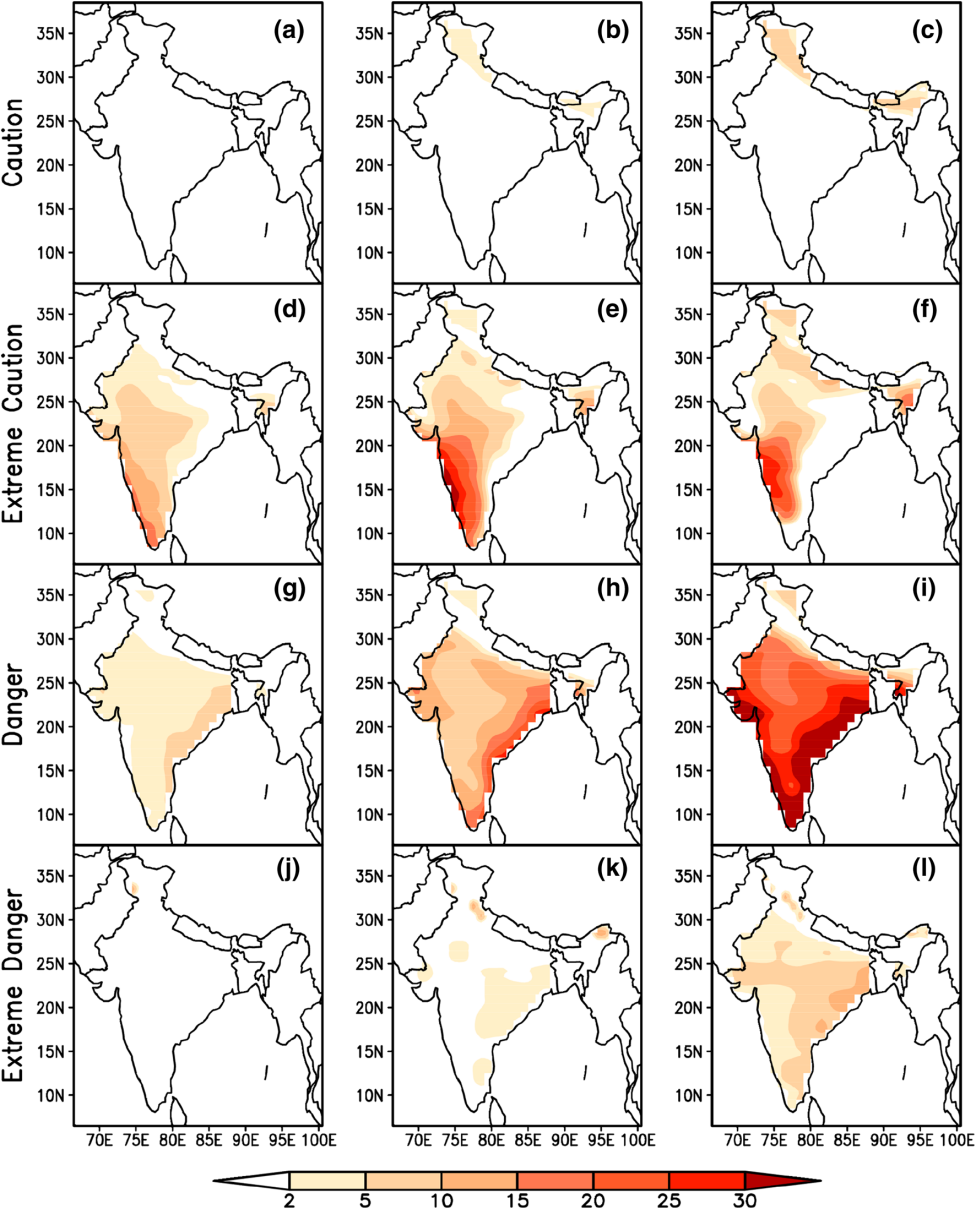
Same as Fig. 2 but for RCP 8.5. This figure has been generated using GrADs 2.1.1.6.0 https://cola.gmu.edu/grads.

Evaluation of summertime mean climatology of heat stress index for the baseline period (1986 to 2005) from CMIP5 Multi Model Mean (MMM) and observed reanalysis data sets along with their biases (model minus observed climatology) revealed the better performance of CMIP5 MMM in capturing the heat stress characteristics over India. Figure 1 indicates that the higher percentage of heat stress days fall in *caution* and *extreme caution* categories in the baseline period. During the four categories of heat stress days (*caution, extreme caution, danger and extreme danger*), the bias is less than 5% which provides a fair confidence on the reliability of model data as these biases are inevitable due to the different physics schemes used by the different GCMs that are being used as multi model mean^61^. Uncertainties in heat stress estimations that include temperature and humidity together are typically lesser than the uncertainties when they used independently^66^. The spatial distribution of projected changes in percentage of days in different categories of heat stress with respect to the base line period during summertime in three times slices (2016–2035, 2046–2065 & 2080–2099) obtained from the CMIP5 MMM of RCP 4.5 & 8.5 are shown in Figs. 2 and 3 respectively. A perceptible increase in the percentage of days falling in *extreme caution* and *danger* categories is observed in both the emission scenarios and is similar to the heat stress trends observed globally for the historical periods by CMIP5 data sets^39^. We found similar to Raymond (2020)^67^ that parts of west coast and east coast are the most affected zones by increased heat stress. An increase of 20 to 30% of days is projected in the *extreme caution* and *danger* categories over these regions during the periods 2046–2065 and 2080–2099 respectively.

**Figure 4:**
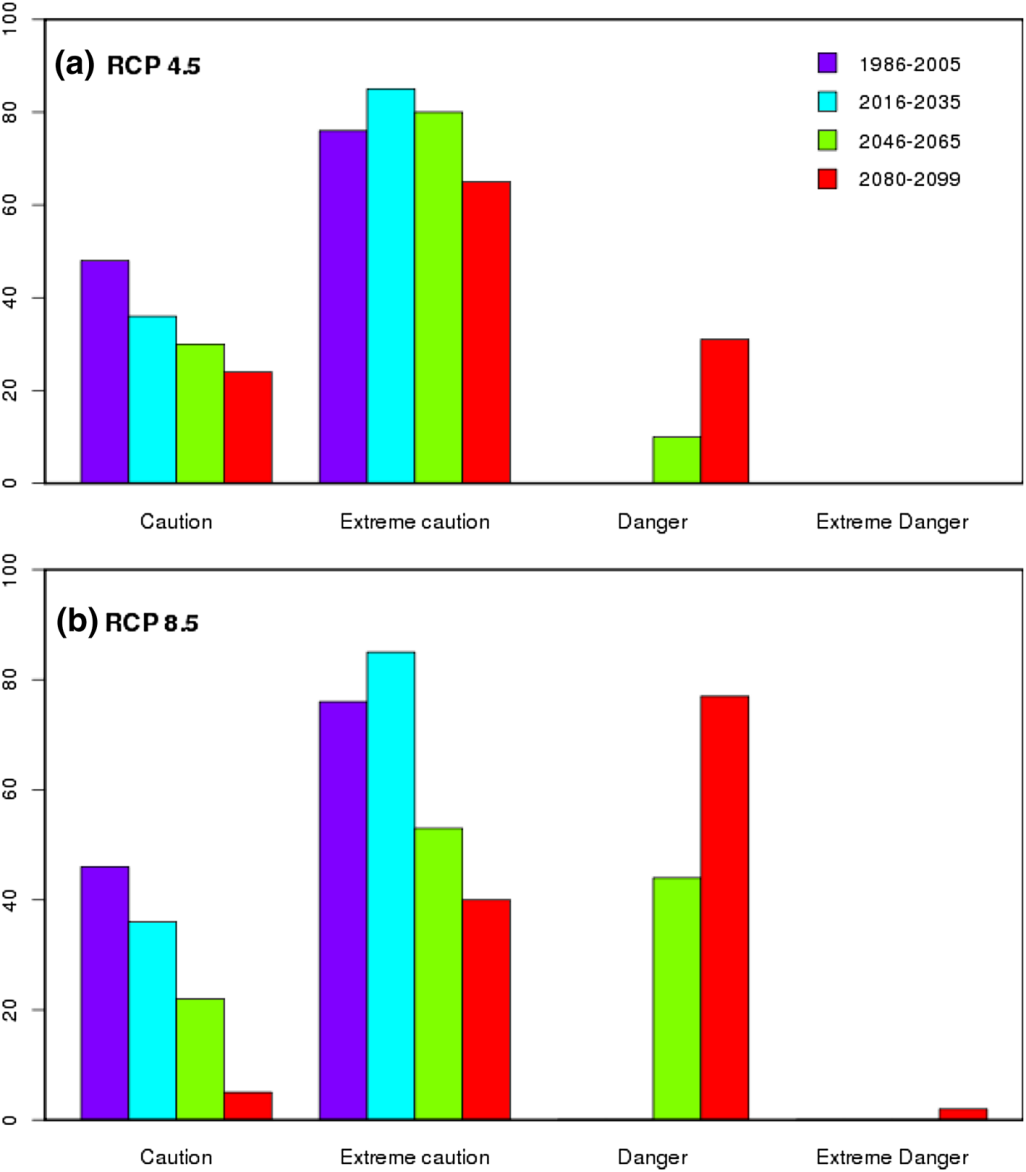
MMM of CMIP5 historical (1986–2005, purple) and future projections for three-epochs ( 2016–2035 (blue), 2046–2065 (green) and 2080–2099 (red) ) of number of heat stress days/year over east coast region under RCP 4.5 (top) & RCP 8.5 (bottom). This figure has been generated using RStudio 1.2.5019 https://rstudio.com/products/rstudio.

**Figure 5:**
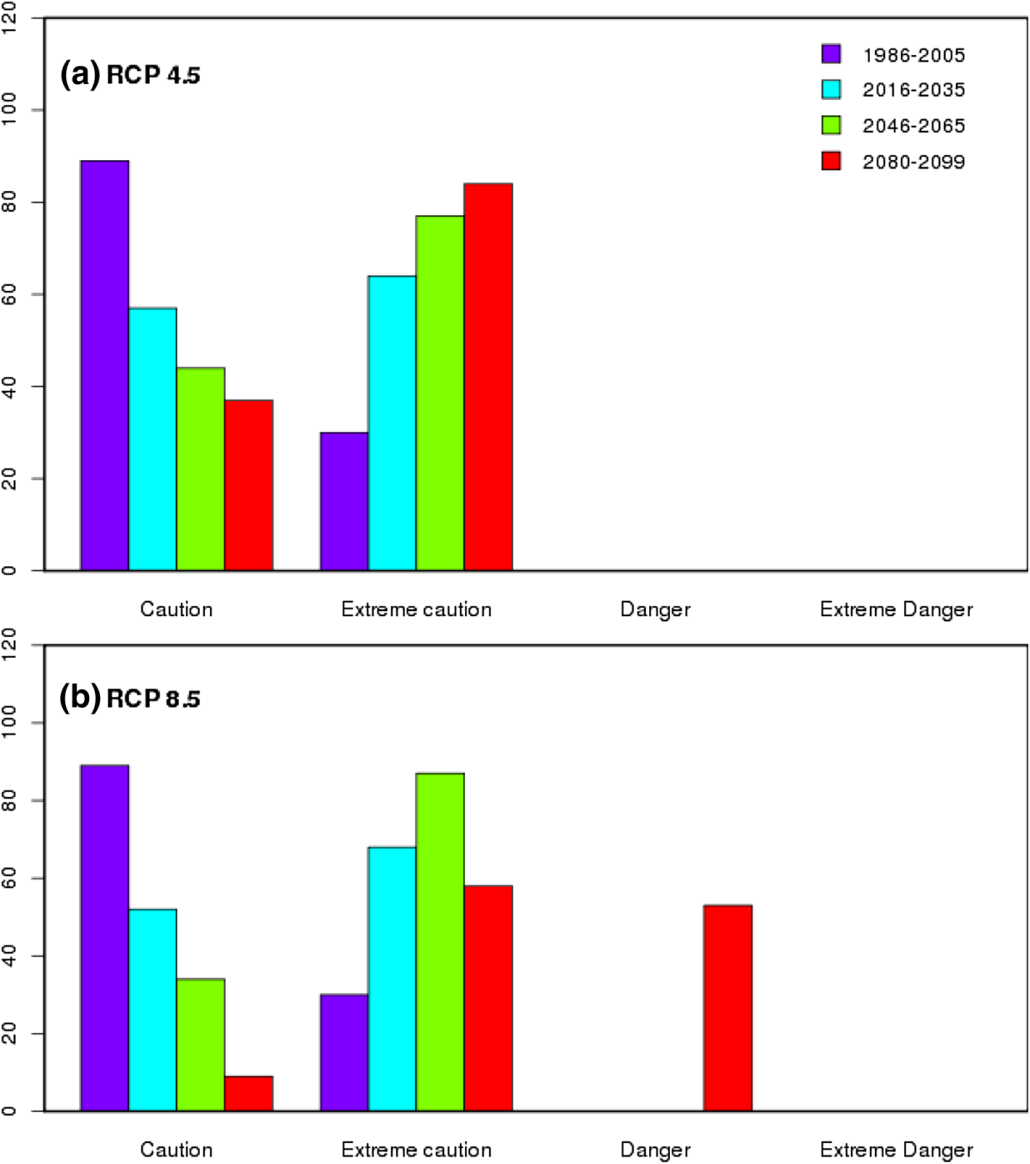
Same as Fig. 4 but for West coast region. This figure has been generated using RStudio 1.2.5019 https://rstudio.com/products/rstudio.

Further, examination of heat stress over east and west coastal regions (upto 200 km interior from the coast) indicated the domination of *extreme caution* days per year in RCP 4.5 & 8.5 followed by *danger* days. The results show that among the two coastal regions, east coast of India may experience a greater number of heat stress days in future under both emission scenarios. Exceptionally over the east coast region, people suffer with heat stress more than 60 days during the summer time of an year under *extreme caution* category while it exceeds 40 days in *danger* category by end of the century under the both emission scenarios (Fig. 4), while the number of days with heat stress are predominantly increasing over west coast (> 32 days) under *extreme caution* category in both the scenarios (Fig. 5). Compared to east coast, west coast is not much vulnerable but both emission scenarios show an increase in extreme *caution* days and project an increase in *danger* days under RCP 8.5 by end of the century. It is reported that the anomalous westerlies over the Indian land mass which reduce the land sea thermal contrast may cause the hot conditions over the eastern coastal regions of India^68^ Significant rise in maximum temperature over the west coast^69^ and east coast^70^ caused the diurnal temperature range of 1–2 °C over the coastal regions for the period 1951 to 2010 which may contribute to the higher loads of heat stress over these areas.

**Figure 6:**
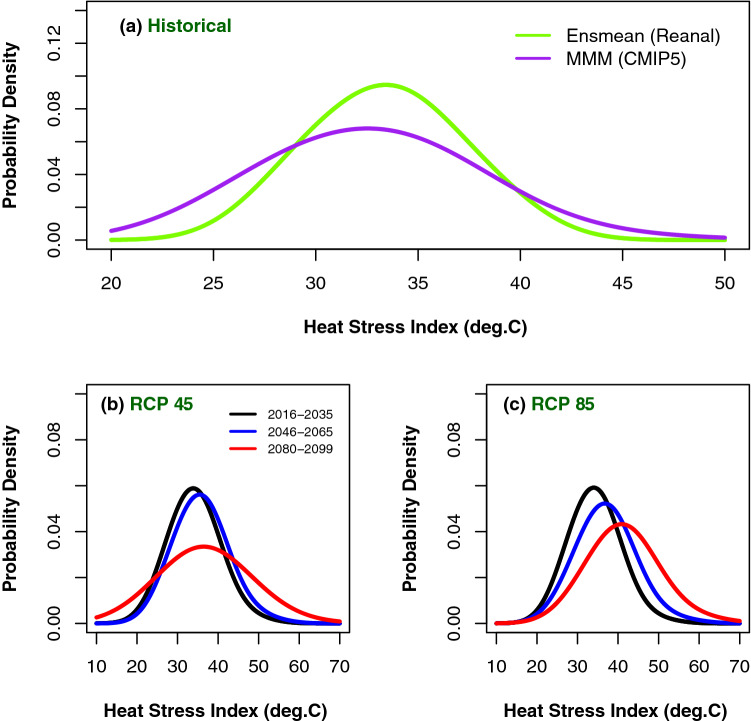
Probability density function (PDF) of heat stress index for (**a**) CMIP5 historical simulations (purple) compared with reanalysis (green) and future three time-epochs under (**b**) RCP 4.5 and (**c**) RCP 8.5 for east coast region. This figure has been generated using RStudio 1.2.5019 https://rstudio.com/products/rstudio.

**Figure 7:**
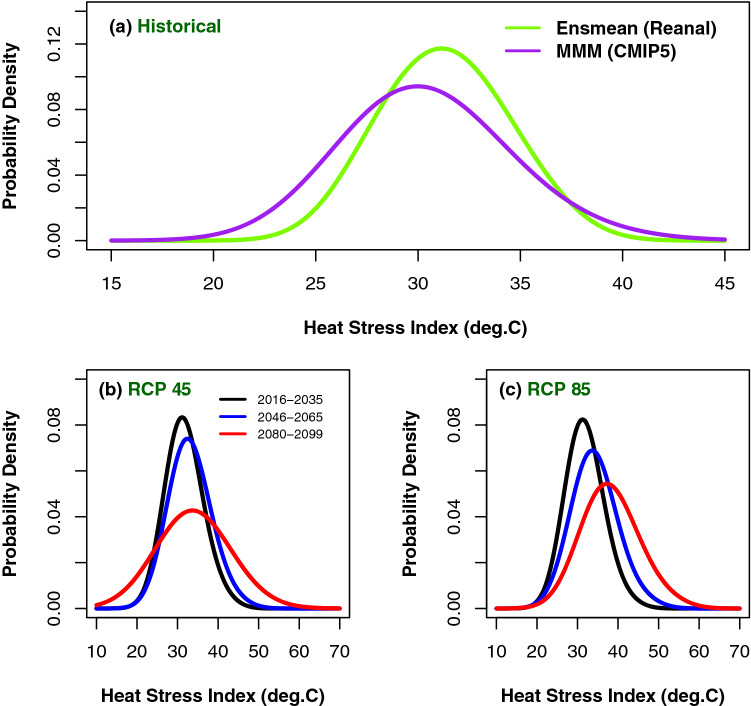
Same as Fig. 6 but for West coast region. This figure has been generated using RStudio 1.2.5019 https://rstudio.com/products/rstudio.

The probability density function (PDF) curves of daily heat stress under RCP 4.5 & 8.5 scenarios over east and west coast regions of India (Figs. 6 and 7) show the positive shifts of location and scale parameters over the east and west coastal regions. Over the east coast region, the maximum probable value of simulated heat stress index is around 34, while towards the end of the century under RCP 4.5 it is around 38 and under RCP 8.5 it is around 42, suggesting that the heat stress is becoming more intense towards the end of the century. Though the curve for the period 2080–2099 is flatter than the other two curves, under both the scenarios, they show the shift in location towards right. Also the probabilities of low heat stress index do not show much difference for all the three time epochs and both the scenarios (left tails of the distribution). However the probability for higher values of heat stress is substantially more towards the end of the century (right tails). The PDFs look almost symmetric but they are positively skewed which clearly reveals that the heat stress index would be more severe (in danger and extreme danger category) in terms of duration and intensity towards the end of the century. Flatness of the curve implies that the kurtosis of the curves are less towards the end of the century i.e., variance or spreads of the distributions are more. Similar features can be observed over west coast region as well. The important point brought out by this analysis is substantial shift of location to the right and increase in spread of the distribution towards the higher values towards the end of the century under both the scenarios. It is clearly seen from the figures (Figs. 6 and 7) that probability of occurrence of danger and extreme danger days over the two coastal regions increase towards the end of the century because of the increase in the duration and intensity of warm days in future. This lengthening of heat stress period and increasing chance of occurrence may result in the occupational health hazards and productivity loses. A similar increase in the heat stress is being reported over different geographical regions of the globe and it has been documented that the trends in heat stress have been more detectable than those temperature^71^.

The increased scenarios of heat stress are mainly due to the increasing temperatures along with the changes in the humidity. The CMIP5 models show warming over India exceeding 5 °C during summer time and this increase in temperature is not consistent within the three time slices under the two climate scenarios (under RCP 4.5 and RCP8.5) (Supp Fig. 1). A slight decrement from the mean has been observed in relative humidity which accounts for more discomfort with the increase in temperature during the twenty-first century (Supp Fig. 2). The mean surface winds at 1000 hPa and sea level pressure (SLP) during summertime for future scenarios have been analyzed to understand the surface circulation during the elevated levels of heat stress. Future surface circulation pattern under RCP 8.5 scenario shows the strengthening of easterlies over Bay of Bengal and south-westerlies over Arabian Sea reaching Indian landmass due to the increased surface pressure gradients which may have possible connection with the increased temperatures in combination with the humid conditions that probes the higher heat stress over the coastal regions (Supp. Fig. 3). As the Bay of Bengal and the Arabian Sea are continued to warm in the recent decades, the higher SSTs cause the evaporative cooling mechanisms of the oceans which transports the winds with the subdued moisture as they reach the Indian land mass^72^. This subdued moist winds in combination with the already raised temperatures over land, together create the higher values of heat stress. The increase in the heat stress is also attributed to the increased anthropogenic forcing, increasing trends of temperature and decreasing trends in humidity during summer time^70^. Also, the air which is heated due to the absorbing aerosols in central and northeastern parts of India sinks in northwestern and southern Indian regions that cause more heating in the recent decades^73^. The CMIP5 MMM projections for the summertime furthermore suggest anomalous south easterlies from Bay of Bengal associated with high pressure may cause high temperatures over the east and west coasts of India. This might favor the enhancement in the severity of heat stress. In other words, the background atmospheric conditions are favorable for the enhanced severity of heat stress particularly by the end of the century.

Prominent features are seen in the upper troposphere like anti-cyclonic circulation over Pakistan region which is intensified towards the end of the century. The future minus present climatology suggests the positive height anomalies and anomalous anti- cyclonic flow at 500 hPa level over northern portions of India (Supp Fig. 4). Anticyclonic conditions over the study area also provide dry moist weather that increases the solar radiation flux due to subsidence of air masses. There is an indication that geopotential height (GPH) gradually strengthens during the future climate scenario. Future climate projections of amplified GPH anomalies at 500-hPa GPH indicate the significant increase of heat waves over India. Positive GPH anomalies at 500 hPa dynamically cause subsidence, clear skies, light winds, warm-air advection, and prolonged hot weather conditions at the surface^4^. Here we also observe a strong positive GPH in the upper troposphere (at 200-hpa) which might be also a causal factor for the surface heat wave and other heat extremes (supp. Figure 5). The possible link between circulation and extreme heat occurrence infers the role of horizontal temperature advection of warm dry air from the interior continental parts to coastal regions as off shore wind direction.

**Figure 8:**
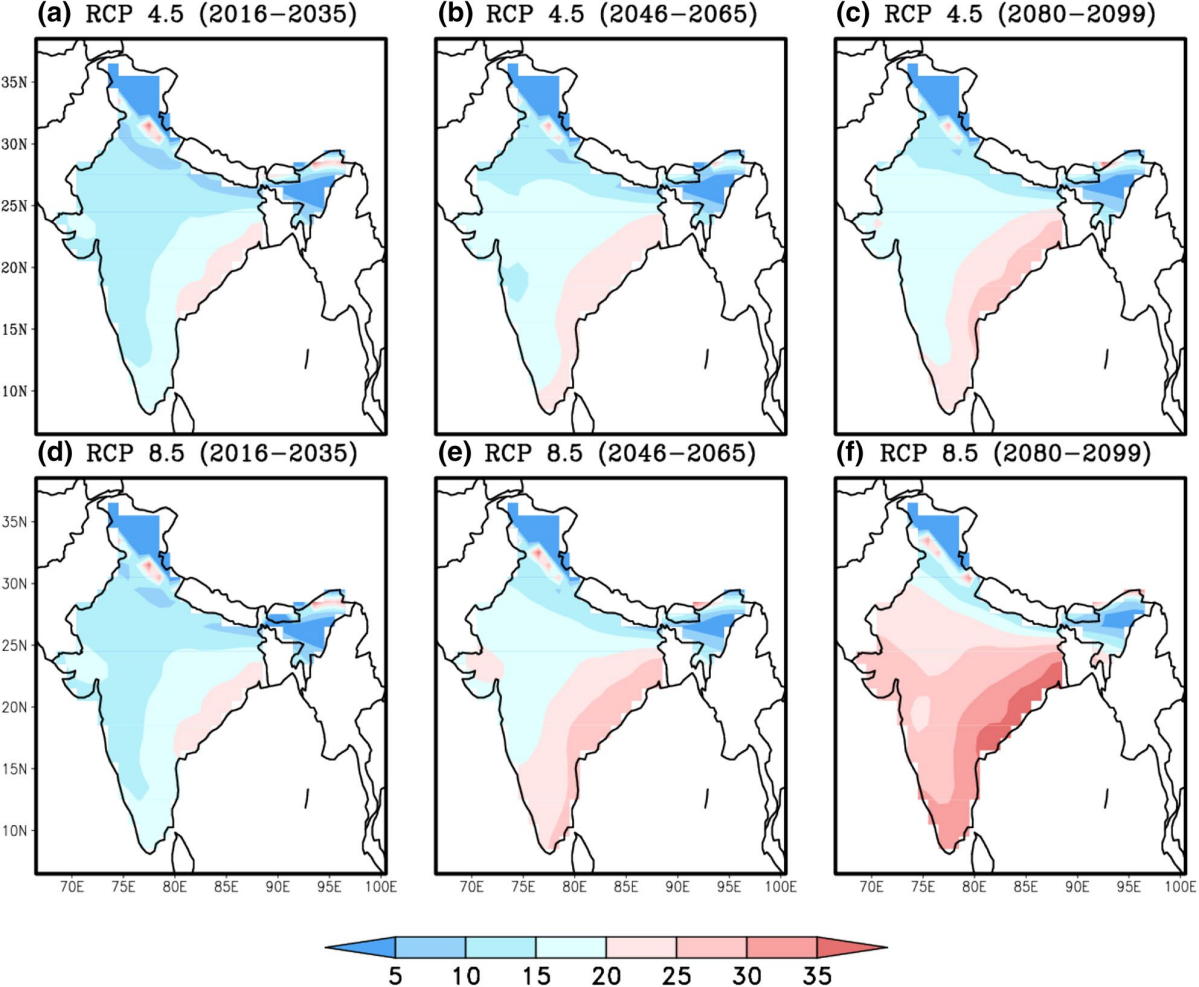
Projected changes in decrements in summertime work performance (%) in three time-epochs of 2016–2035, 2046–2065 and 2080–2099 under RCP 4.5 (top) & RCP 8.5 (bottom). This figure has been generated using GrADs 2.1.1.6.0 https://cola.gmu.edu/grads).

Climate change leaves the workers in lurch who work under extreme climates^74^. The heat stress is projected to intensify health risks of population and decrease the labour capacity, particularly over tropical and sub-tropical areas^75,76^. The decline in work performance over India is conspicuous during 2046–2065 and 2080–2099 than during 2016–2035 (Fig. 8). The cognitive fatigue, difficulties in mental concentration which reduces the work efficiency due to rise in temperature may result in the productivity losses^77^. In addition, the sweat evaporation stops at higher relative humidity values which hinder the cooling effect of the body leading to heat illness^78^. The climate models have shown the heat stress vulnerability in the regions of Southeast Asia, southeastern US and Northern Australia^6^. Dunne et al.^75^ proposed a fit for estimating labor capacity and reported a reduction in labor work capacity upto 40% under RCP 8.5 scenario over tropical and mid latitudinal region and also found that India will undergo heat stress more quantitatively than Eurasia and greater Caribbean region. The simulated levels of moist heat stress obtained from RCP 8.5 show the substantial rise in wet bulb temperature extremes over India, China, Southeast Asia, interior South America, and western Africa^79^. The urban heat island effect caused by the heat and moisture from the concrete structures that affects the incoming and outgoing solar radiation also has an impact on the heat stress in urban areas^80^. The impacts of climate change on working performance in India further worsen in view of RCP 8.5 scenario where India is likely to experience 3 to 4 degrees of warming^81^. The decrement in the work performance is highly susceptible in the southeastern coastal regions of India under the RCP 4.5 scenario while the same extends to total country except in the belt of Himalayas under the RCP 8.5 scenario. Our assessment showed a quantitative decline in work performance to up to 40% under the RCP8.5 and 35% under the RCP 4.5 scenario. The initial decline in work performance is conspicuous over the east coastal regions of India and later expanded to most of the regions in the country by end of the century. As India witnesses the notorious climate change impacts over the coastal regions, where 250 million people live within 50 km of the coast line^82^, the decline in work performance will be a major threat to the livelihood of the coastal communities’. However, a rigorous evaluation of work performance needs to be done based on individual and population-based characteristics. We provide here a common understanding in the projected decreasing levels of work performance as an overall assessment though the parameters such as local work place conditions^83^, construction of buildings^84^, clothing^85^ and physical work intensity^86^ have not been considered as input parameters. As suggested by Brode et al.^44^, Dunne et al.^75^ and Buzan and Huber^79^, estimation of the work performance needs to be contextualized and should elevate the role of changing the duty hours of labor, type of the labor. Our effort in the present study is to report the general features of declining work performance under future warm and humid conditions under global warming scenario. Also, the present study considers the mean temperature for estimation of heat stress that will provide a fair understanding of work performance. However, the diurnal cycle of the temperature may also play a key role under changing work hours of the labor. Finally, the practical implications of these elevated heat stress ‘*need the support of engineering, social and policy-making decisions’*^79^.

## Conclusions

Heat stress is expected to intensify, due to unprecedented warming during the twenty-first century. The increasing levels of heat stress pose a great challenge to mitigate and to adapt proper mechanisms to confront it. Decline in work performance associated with the detectable trends of heat stress over India point out the dire need to frame the sage work procedures, preventing the heat-based illness to reduce the health hazards as well as to maintain the work performance. The coastal regions of India (east and west coast) are found to be more vulnerable to heat stress impacts by showing a perceptible increase in the notorious impact days and a decline of 30 to 40% in the work performance, particularly in east coast region.

## Supplementary information

Supplementary information.
