# Supplementary material for: Projections of heat stress and associated work performance over India in response to global warming
Source: Sci Rep. 2020 Oct 7;10:16675. doi: 10.1038/s41598-020-73245-3 (PMC7542441; doi:10.1038/s41598-020-73245-3)

**Projections of heat stress and associated work performance over India in response to global warming**

K. Koteswara Rao^1^, **T.V. Lakshmi Kumar^2*^, Ashwini Kulkarni^1^, Chang – Hoi Ho^3^, B. Mahendranath^4^, Srinivas Desamsetti^5^, Savita Patwardhan^1^, Appala Ramu Dandi^1^, Humberto Barbosa^6^, Sudhir Sabade^1^**

^1^. Indian Institute of Tropical Meteorology, **Ministry of Earth Sciences, India**.

**^2*^Atmospheric Science Research Laboratory, SRM Institute of Science and Technology, Kattankulathur,Tamilnadu,India.**

**^3^School of Earth and Environmental Sciences, Seoul National University, Seoul, South Korea.**

**^4^Department of Meteorology & Oceanography, Andhra University, Visakhapatnam, India.**

**^5^ National Centre for Medium Range Weather Forecasting, Ministry of Earth Sciences, India.**

^6^Laboratorio de Analise e Processamento de Imagens de Satelites, Universiadade Federal de Alogoas- UFAL, Maceió, Brazil

**lkumarap@hotmail.com**

**Supplementary Material**

**Supplementary figure illustrations:**

Supp. Fig. 1 Simulations of summertime (MAMJ) temperature anomalies by individual CMIP5 models (gray), MMM mean (black) compared with reanalysis mean (green) for 1986-2005 and projected temperature anomalies under RCP 4.5 by individual models (cyan) and MMM (blue) as well as RCP 8.5 individual (orange) and MMM mean (red) for 2006-2100. This figure has been generated using Xmgrace 5.1.24 <http://exciting-code.org/xmgrace-quickstart>.

Supp. Fig.2 Same as Supp. Figure.1 but for relative humidity (%).This figure has been generated using Xmgrace 5.1.24 <http://exciting-code.org/xmgrace-quickstart>.

Supp. Figure.3: Project changes in sea level pressure (hpa) and surface winds at 1000 hpa (m/s) under RCP 8.5 with respect to 1986-2005 for three-time epochs 2016-2035 (top), 2046-2065 (middle) an 2080-2099 (bottom). This figure has been generated using GrADs 2.1.1.6.0 [http://cola.gmu.edu/grads](http://cola.gmu.edu/grads/)

Supp. Fig.4 Same as supp. Figure.3 but for Geopotential height and vector winds at 500hpa. This figure has been generated using GrADs 2.1.1.6.0 [http://cola.gmu.edu/grads](http://cola.gmu.edu/grads/)

Supp. Fig.5 Same as supp. Figure.4 but for Geopotential height and vector winds at 200hpa. This figure has been generated using GrADs 2.1.1.6.0 [http://cola.gmu.edu/grads](http://cola.gmu.edu/grads/)


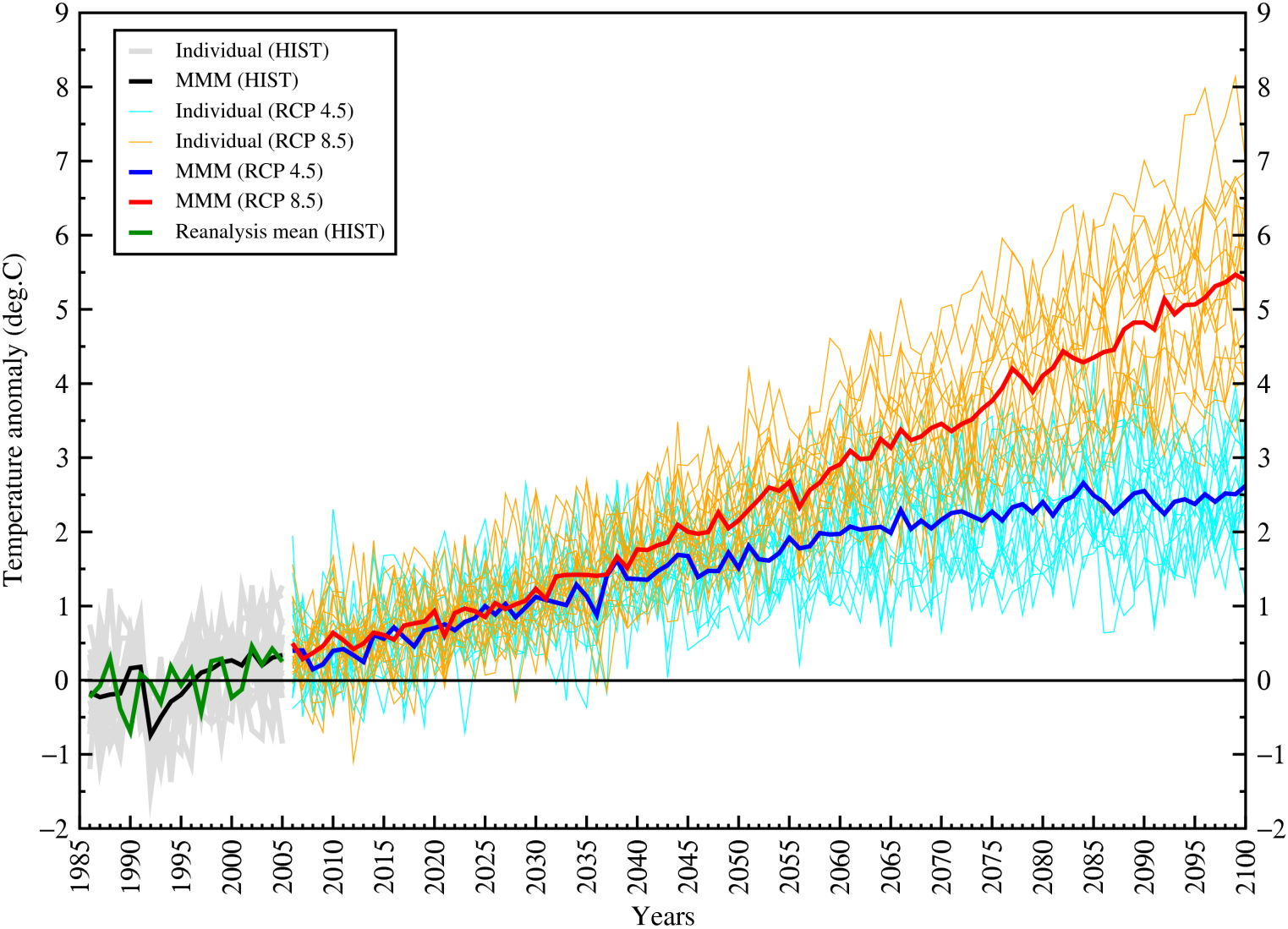


Supp. Fig. 1 Simulations of summertime (MAMJ) temperature anomalies by individual CMIP5 models (gray), MMM mean (black) compared with reanalysis mean (green) for 1986-2005 and projected temperature anomalies under RCP 4.5 by individual models (cyan) and MMM (blue) as well as RCP 8.5 individual (orange) and MMM mean (red) for 2006-2100. This figure has been generated using Xmgrace 5.1.24 <http://exciting-code.org/xmgrace-quickstart>.


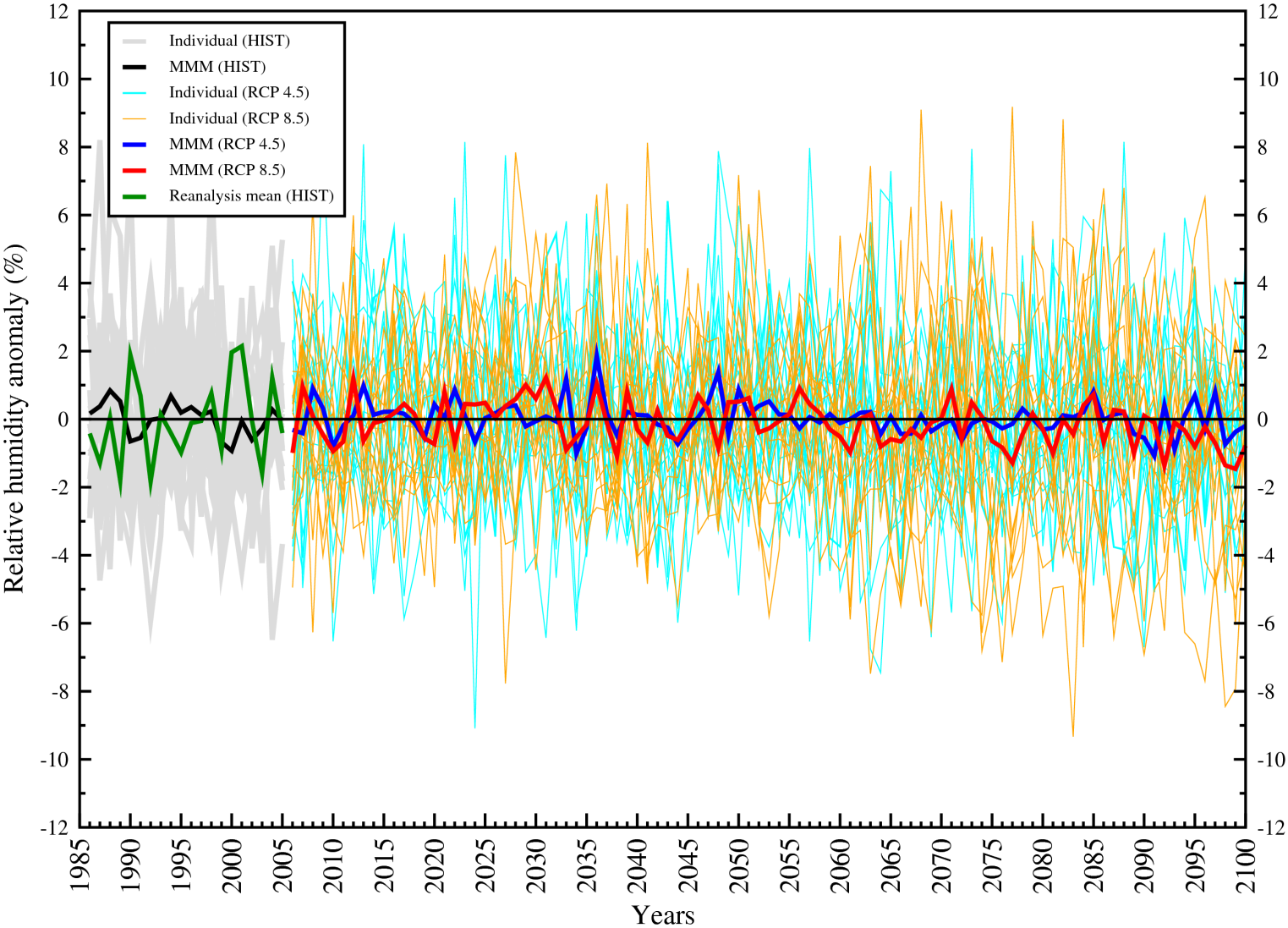


Supp. Fig.2 Same as Supp. Figure.1 but for relative humidity (%).This figure has been generated using Xmgrace 5.1.24 <http://exciting-code.org/xmgrace-quickstart>.


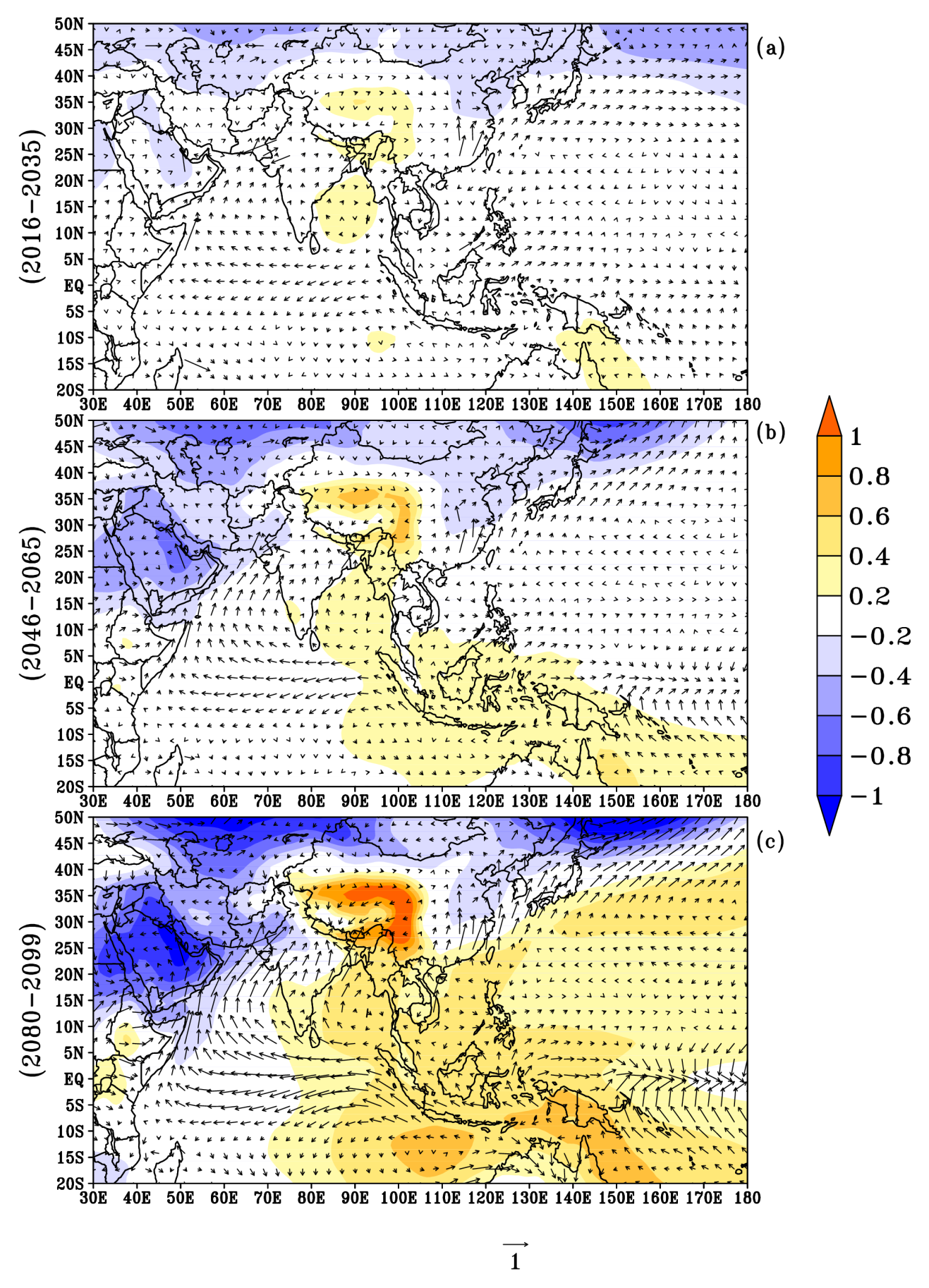


Supp. Figure.3: Project changes in sea level pressure (hpa) and surface winds at 1000 hpa (m/s) under RCP 8.5 with respect to 1986-2005 for three-time epochs 2016-2035 (top), 2046-2065 (middle) an 2080-2099 (bottom). This figure has been generated using GrADs 2.1.1.6.0 [http://cola.gmu.edu/grads](http://cola.gmu.edu/grads/)


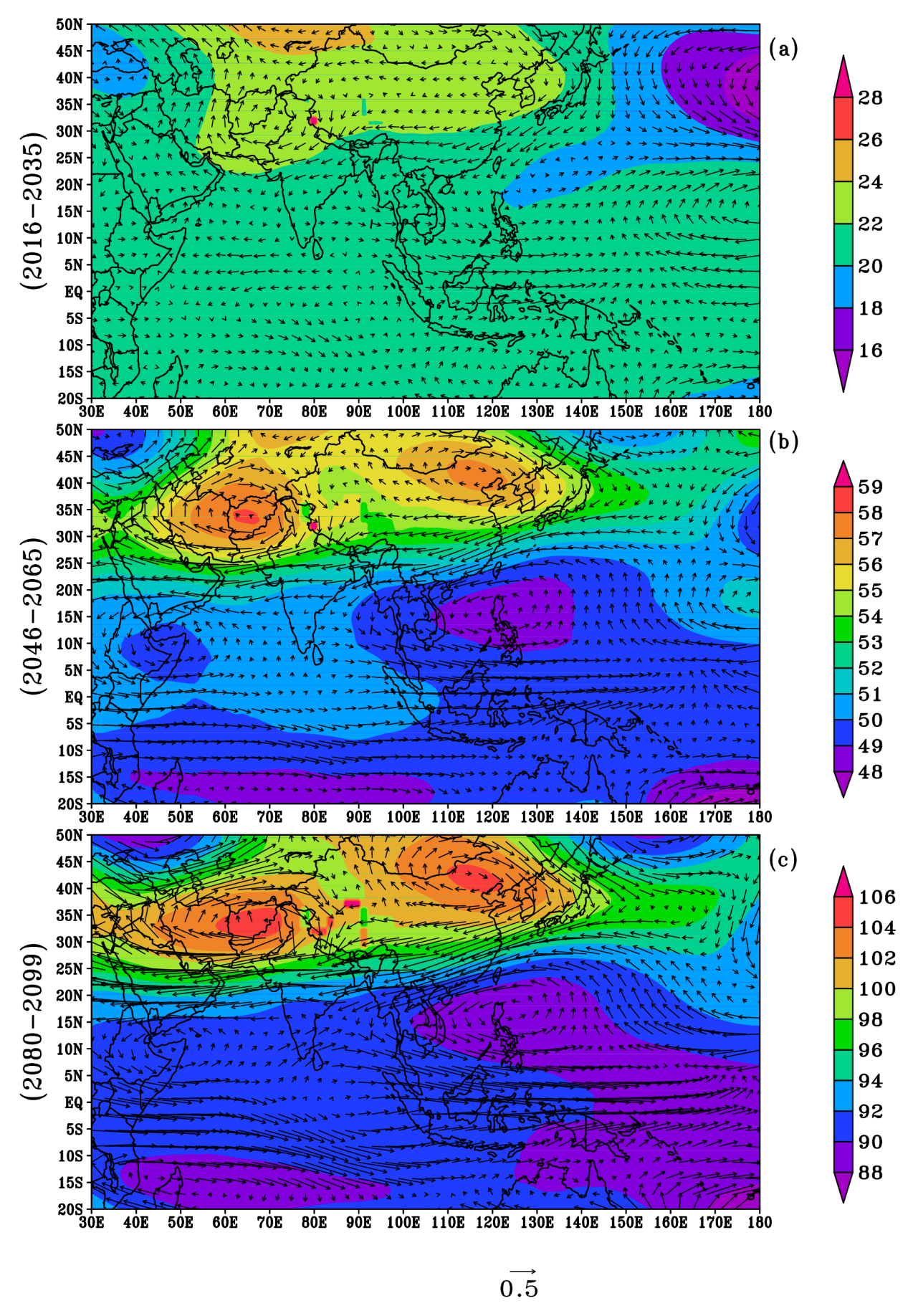


Supp. Fig.4 Same as supp. Figure.3 but for Geopotential height and vector winds at 500hpa. This figure has been generated using GrADs 2.1.1.6.0 [http://cola.gmu.edu/grads](http://cola.gmu.edu/grads/)


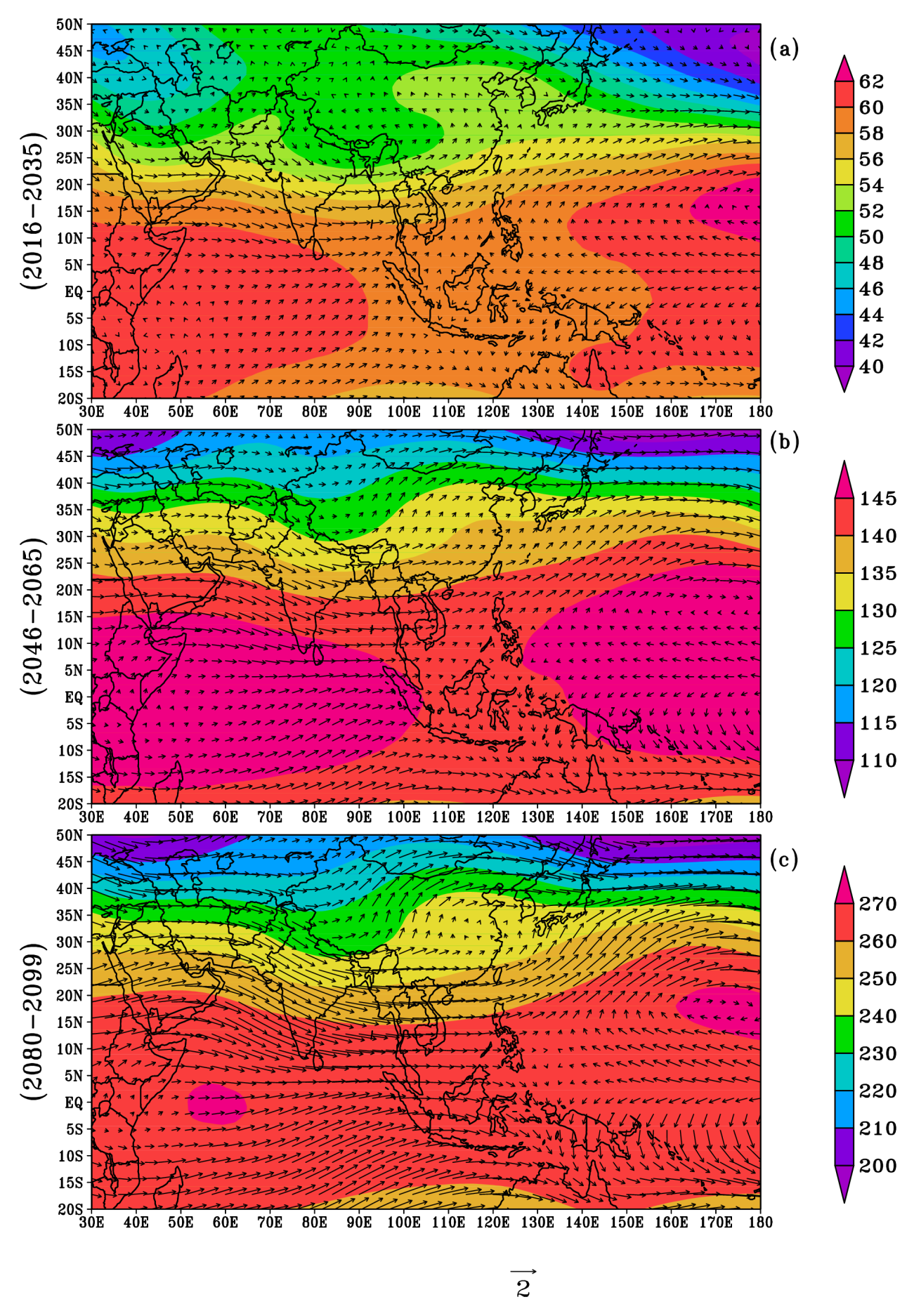


Supp. Fig.5 Same as supp. Figure.4 but for Geopotential height and vector winds at 200hpa. This figure has been generated using GrADs 2.1.1.6.0 [http://cola.gmu.edu/grads](http://cola.gmu.edu/grads/)

**Details on the evaluation of HI:**

The Heat Index (Steadman, 1979, 1984) proposed by Steadman is designed based on the several earlier bio-meteorological studies using the five empirical models (Schoen, 2005). The formula is a multi regression model which takes into account air temperature and humidity. While developing the formula, Steadman considered the parameters such as people activity, clothing etc., He used the two aforementioned variables and converted into a single formula that is measured in the units of air temperature. The formula basically illustrates the current weather conditions that would be felt by the humans when the dew point temperature is 14 degree Celsius.

In order to arrive at an equation which uses more conventional independent variables, a multiple regression analysis was performed on the data from Steadman's table. The resulting equation could be considered a Heat Index equation (Rothfusz, 1990)

**HI = -42.379 + 2.04901523T + 10.14333127R – 0.22475541TR – 6.83783 × 10-3T2**

**-5.481717×10-2R2 + 1.22874×10-3 T2R + 8.5282×10^-4^ TR2 – 1.99×10^-6^T2R2 (Eq. 1)**

Where,

T = ambient dry bulb temperature

R = relative humidity

This equation is obtained by multiple regression analysis, the heat index value (HI) has an error of ±1.3°F (1.5ºC or 274.65K). The values of heat index were then further converted in Celsius scale.


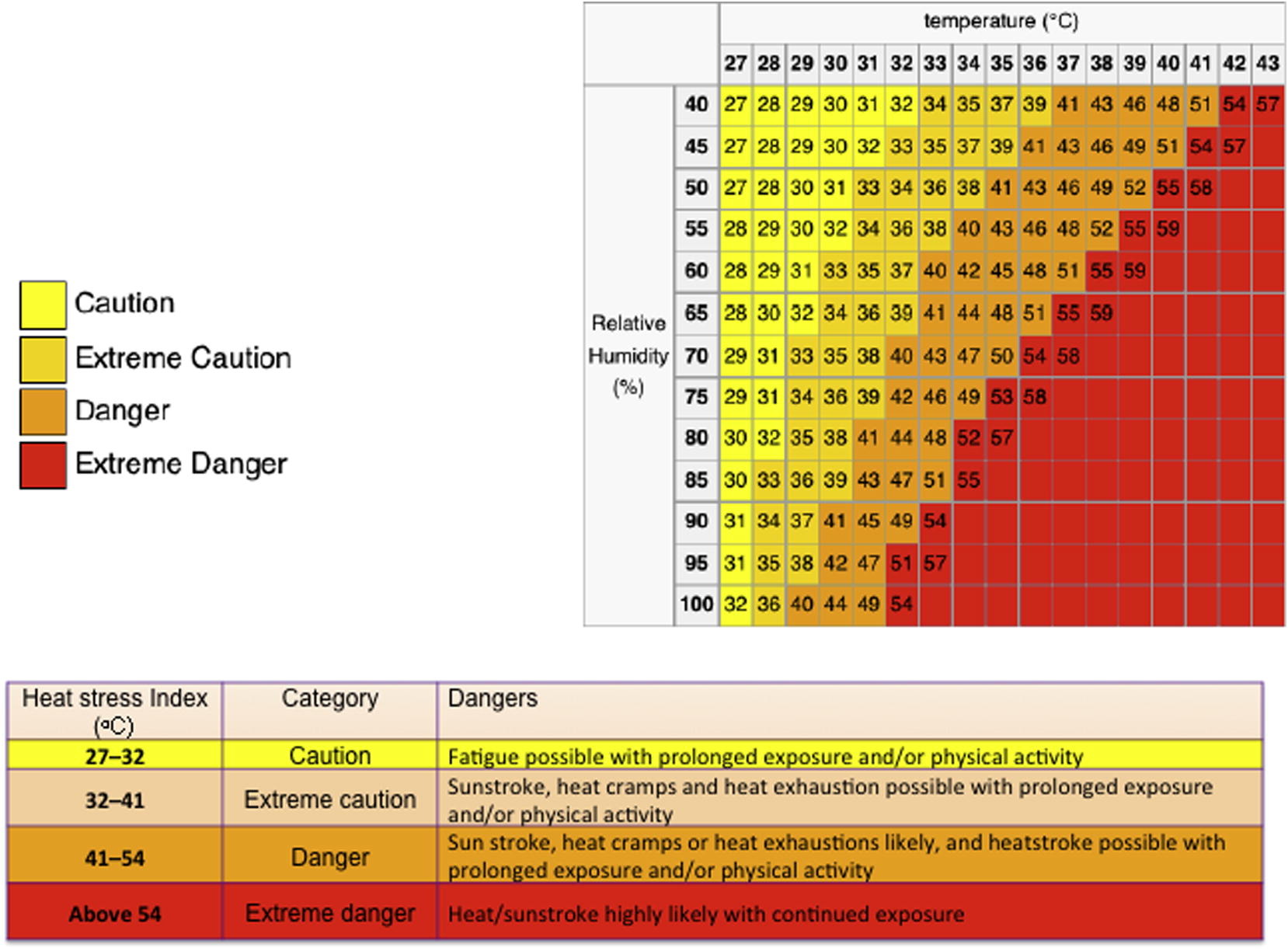

Supplement: Supplementary file 1 — Supplementary information. [file 41598_2020_73245_MOESM1_ESM.doc]
